# Contribution of H3K4 demethylase KDM5B to nucleosome organization in embryonic stem cells revealed by micrococcal nuclease sequencing

**DOI:** 10.1186/s13072-019-0266-9

**Published:** 2019-04-02

**Authors:** Jiji T. Kurup, Ion J. Campeanu, Benjamin L. Kidder

**Affiliations:** 1 Department of Oncology, Wayne State University School of Medicine, Detroit, MI USA; 2 Karmanos Cancer Institute, Wayne State University School of Medicine, Detroit, MI USA

**Keywords:** Embryonic stem cells, Pluripotent, Epigenetics, Micrococcal nuclease, MNase, Nucleosome positioning, Chromatin, ChIP-Seq, KDM5B, H3K4me3

## Abstract

**Background:**

Positioning of nucleosomes along DNA is an integral regulator of chromatin accessibility and gene expression in diverse cell types. However, the precise nature of how histone demethylases including the histone 3 lysine 4 (H3K4) demethylase, KDM5B, impacts nucleosome positioning around transcriptional start sites (TSS) of active genes is poorly understood.

**Results:**

Here, we report that KDM5B is a critical regulator of nucleosome positioning in embryonic stem (ES) cells. Micrococcal nuclease sequencing (MNase-Seq) revealed increased enrichment of nucleosomes around TSS regions and DNase I hypersensitive sites in KDM5B-depleted ES cells. Moreover, depletion of KDM5B resulted in a widespread redistribution and disorganization of nucleosomes in a sequence-dependent manner. Dysregulated nucleosome phasing was also evident in KDM5B-depleted ES cells, including asynchronous nucleosome spacing surrounding TSS regions, where nucleosome variance was positively correlated with the degree of asynchronous phasing. The redistribution of nucleosomes around TSS regions in KDM5B-depleted ES cells is correlated with dysregulated gene expression, and altered H3K4me3 and RNA polymerase II occupancy. In addition, we found that DNA shape features varied significantly at regions with shifted nucleosomes.

**Conclusion:**

Altogether, our data support a role for KDM5B in regulating nucleosome positioning in ES cells.

**Electronic supplementary material:**

The online version of this article (10.1186/s13072-019-0266-9) contains supplementary material, which is available to authorized users.

## Background

Nucleosomes represent the basic repeating structural unit of chromatin [1, 2], where 146 base pairs (bp) of DNA are wrapped around an octamer of histones. Nucleosomes constitute the first level of compaction of DNA into a chromatin structure in the nucleus of eukaryotic cells, and the organization of nucleosomes influences gene activity in part by controlling chromatin accessibility. Nucleosomes are depleted at transcriptional start sites (TSS), and arrays of nucleosomes are positioned adjacent to nucleosome-depleted regions [3, 4]. Covalent posttranslational modifications of histone *N*-terminal tails, such as methylation, are instrumental in regulating gene expression. Trimethylation of histone 3 lysine 4 (H3K4me3) is primarily localized at TSS of active genes [5–9], where it functions in part as a target for gene activation [10–12]. Lysine demethylase 5 (KDM5) family members, such as KDM5B, remove H3K4 methylation [13, 14]. KDM5B plays fundamental roles in development [15, 16], ES cell differentiation [17–19], and trophoblast stem (TS) cell differentiation [20]. KDM5B and H3K4me3 co-localize at promoters of active genes in ES cells, where KDM5B focuses H3K4me3 near promoters to prevent it from spreading to gene bodies [18], an effort that may support transcriptional consistency by resetting H3K4 methylation after the transcriptional cycle by demethylating gene body regions. KDM5B performs a similar function to focus H3K4 methylation during mouse embryonic preimplantation stage development, where KDM5B demethylates broad H3K4me3 domains to prevent an increase in lengthening of H3K4me3 domains [21]. While these results demonstrate that KDM5B regulates H3K4 methylation at promoter and gene body regions, it is unclear how KDM5B controls nucleosome organization and chromatin accessibility in ES cells. Moreover, studies have demonstrated that nucleosome distributions are influenced by transcription initiation and elongation [22, 23], processes which are controlled in part by KDM5B [18, 24]. Therefore, to clarify the role for KDM5B in regulating nucleosome organization in ES cells, we evaluated genome-wide changes in nucleosome positioning in KDM5B-depleted and control ES cells using micrococcal nuclease sequencing (MNase-Seq). Our findings demonstrate that depletion of KDM5B leads to altered enrichment of nucleosomes around TSS regions and accessible chromatin regions (DNase I hypersensitive sites). We also demonstrate that depletion of KDM5B leads to a redistribution and disorganization of nucleosomes, in a DNA sequence-dependent manner. We also uncovered non-canonical nucleosome phasing patterns and asynchronous nucleosome spacing surrounding TSS regions in KDM5B-depleted ES cells. Alterations in nucleosome distributions are correlated with dysregulated gene expression. In addition, we demonstrate that DNA shape features are significantly different at regions with shifted nucleosomes. Our data demonstrate a novel role for KDM5B in controlling nucleosome distributions in ES cells.

## Results

### Evaluation of nucleosome positioning in KDM5B-depleted ES cells

**Fig. 1:**
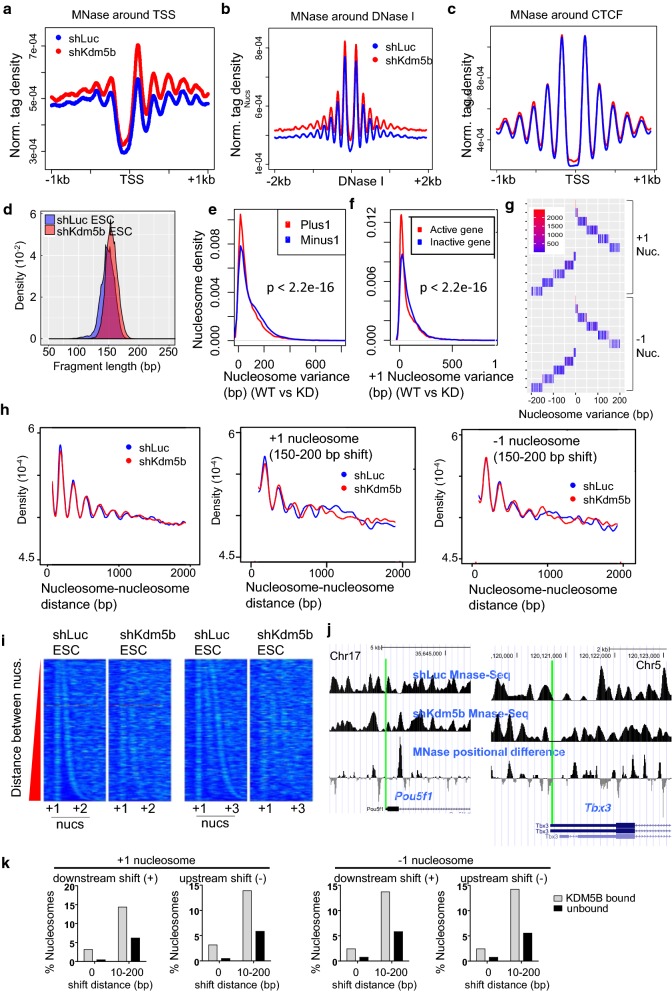
Profiling of nucleosome distributions in KDM5B-depleted ES cells. **a**–**c** Average profile of nucleosome density relative to **a** transcriptional start sites (TSS), **b** DNase I hypersensitive sites, and **c** CTCF-bound regions in KDM5B-depleted and control ES cells. **d** Density plot of nucleosome fragment length (bp) in control (shLuc) and KDM5B-depleted ES cells. **e**, **f** Density plot of nucleosome variance for **e** + 1 nucleosomes (red) or − 1 nucleosomes (blue; relative to TSS) and at **f** active (red; RPKM > 1) and inactive (blue; RPKM < 1) genes (*x*-axis: nucleosome variance (bp); *y*-axis: nucleosome density). **g** Heatmap of nucleosome variance (shift distance in bp) around + 1 nucleosomes and − 1 nucleosomes with respect to TSSs in KDM5B-depleted ES cells. **h** Density plot of nucleosome-to-nucleosome distance nearby active gene promoters (left), and at + 1 or − 1 variant nucleosomes (middle, right). **i** Heatmap of nucleosome densities in shLuc and shKdm5b ES cells sorted by distance between + 1 and + 2 nucleosomes (relative to TSS; left) and between + 1 and + 3 nucleosomes (right). Note the disorganized nucleosome array in KDM5B-depleted ES cells relative to control ES cells. **j** UCSC genome browser view of shLuc and shKdm5b MNase-Seq and MNase-Seq position difference in KDM5B-depleted and control ES cells. For MNase-Seq positional difference, if nucleosome occupancy is greater in shKdm5b ES cells relative to shLuc ES cells, the value is − log10 *P*-value, else, it is log10 *P*-value. Green line depicts TSS. **k** Percentage of KDM5B-bound (gray) and unbound (black) nucleosomes with uniform nucleosome placement (0-bp shift) or shifted + 1 or − 1 nucleosomes (10–200-bp shift) relative to TSS in KDM5B-depleted ES cells

To understand the role for KDM5B in nucleosome organization, we explored genome-wide nucleosome positioning in KDM5B-depleted and control ES cells. KDM5B knockdown (shKdm5b) and control (shLuc) ES cells, which we described previously [17, 18], were stably selected in the presence of 1 µg/mL puromycin. Depletion of KDM5B resulted in > 95% reduction of mRNA as evaluated using Q-RT-PCR (Additional file 1: Fig. S1) [17], which was confirmed using western blotting [17]. Moreover, RNAi depletion of KDM5B resulted in global increases in H3K4me3 and H3K4me2 levels as evaluated using western blotting [17], which is consistent with the role of the H3K4 demethylase KDM5B. Next, we performed MNase digestion of chromatin from KDM5B-depleted and control ES cells and subsequently isolated mononucleosome-sized DNA fragments. We then sequenced the ends using paired-end sequencing and mapped to the mouse genome (see “Methods” section). To interrogate nucleosome distributions around TSS regions, we computed the average distribution of fragments. These findings reveal a marked increase in nucleosome density at TSS regions and surrounding phased nucleosomes across the genome in KDM5B-depleted ES cells relative to control ES cells (Fig. 1a). Consistent with previous findings, we observed depletion of nucleosomes at TSS regions [25–27] for both KDM5B-depleted and control ES cells. We also surveyed nucleosome occupancy around DNase I hypersensitive sites (DHS), which are known to be correlated with transcriptionally active promoters [28], and also identified increased enrichment of nucleosomes around DNase I sites (ENCODE: GSM1014154) in KDM5B-depleted ES cells (Fig. 1b). However, nucleosome enrichment around binding sites of the genomic insulator, CTCF [29], exhibited only nominal increases in enrichment in KDM5B-depleted ES cells (Fig. 1c), suggesting that alterations in nucleosome occupancy in KDM5B-depleted ES cells mainly occur nearby TSS regions and accessible chromatin regions (e.g., DHS).

Sequence reads revealed an altered nucleosome size distribution in KDM5B-depleted ES cells (Fig. 1d). In addition, nucleosome positioning varied significantly at TSS regions between control and KDM5B-depleted ES cells (Fig. 1e). To calculate variation in nucleosome positioning, we measured the mean value of distances between + 1 or − 1 nucleosomes in shLuc and shKdm5b ES cells relative to TSS regions. Results from these analyses highlight significant variation in nucleosome positioning at + 1 and − 1 nucleosomes (Fig. 1e), where nucleosomes are phased relative to TSS regions at active genes. In addition, nucleosome variation was lower at active genes (RPKM > 1) relative to inactive genes (RPKM < 1) in KDM5B-depleted ES cells (Fig. 1f). Danpos was further used to evaluate nucleosome position shifts and occupancy changes using a uniform statistical framework [30]. To further characterize nucleosome variation in KDM5B-depleted ES cells, we stratified nucleosomes by the degree of variation between control and KDM5B-depleted ES cells (− 200 to + 200 bp) into groups of + 1 and − 1 nucleosomes relative to TSS regions that shift upstream or downstream (Fig. 1g). We then evaluated the degree of uniformity and spacing between all nucleosomes on a genome-wide scale and observed relatively similar results for control and KDM5B knockdown ES cells (Fig. 1h, left). However, an evaluation of + 1 or − 1 nucleosome arrays relative to TSSs stratified by shift distance in KDM5B-depleted ES cells (e.g., 150–250 bp) revealed altered uniformity and spacing (Fig. 1h, middle, right). Heatmaps of nucleosome density, sorted by the distance between + 1 and + 2 nucleosomes in control ES cells, also revealed variation in spacing of the + 1 nucleosome between control and KDM5B-depleted ES cells (Fig. 1i). In addition, UCSC custom browser views confirmed variation in nucleosome positioning between control and KDM5B-depleted ES cells at representative pluripotency regulators (Fig. 1j). To investigate a relationship between KDM5B binding and nucleosome shift distance in control and KDM5B-depleted ES cells, we analyzed our KDM5B ChIP-Seq data [18]. These results revealed that + 1 or − 1 nucleosomes bound by KDM5B are more sensitive to shifting relative to regions without KDM5B binding (Fig. 1k). For − 1 nucleosomes that shifted downstream or upstream, we observed 2.4-fold or 2.6-fold more − 1 nucleosomes, respectively, bound by KDM5B relative to unbound nucleosomes (Fig. 1k). In addition, for + 1 nucleosomes that shifted downstream or upstream, we observed 2.3-fold or 2.4-fold more nucleosomes, respectively, bound by KDM5B relative to unbound nucleosomes (Fig. 1k). These findings suggest that KDM5B binding nearby TSSs is correlated with a redistribution of nucleosomes in KDM5B-depleted ES cells.

**Fig. 2:**
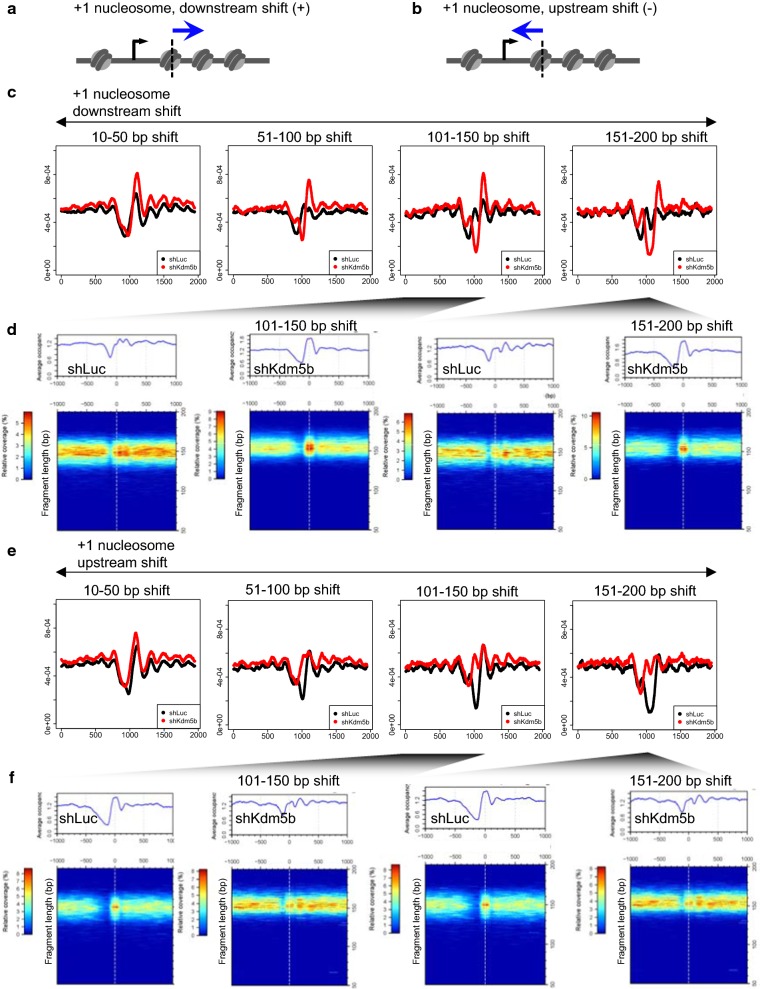
Characterization of altered nucleosome distributions in KDM5B-depleted ES cells. Schema of + 1 nucleosomes relative to transcriptional start sites (TSS) shifted **a** downstream or **b** upstream. **c** Average density profiles of nucleosomes around TSS of genes with + 1 nucleosomes shifted downstream (shift distance: 10–50, 51–100, 101–150, 151–200 bp). Note the altered nucleosome densities and asynchronous array of nucleosomes in KDM5B-depleted ES cells. **d** Two-dimensional (2D) occupancy plot of nucleosomes around TSS of genes with + 1 nucleosomes shifted downstream in KDM5B-depleted ES cells. Plot2DO [31] was used to show relative nucleosome occupancy and fragment length in a heatmap matrix. Average profile of nucleosome occupancy is shown above the heatmap plot. *X*-axis: position relative to center of nucleosome; *y*-axis: fragment length (bp). **e** Average profiles of nucleosomes around TSS of genes with + 1 nucleosomes shifted upstream in KDM5B-depleted ES cells. **f** Two-dimensional (2D) occupancy plot of nucleosomes around TSS of genes with + 1 nucleosomes shifted upstream in KDM5B-depleted and control ES cells

We further explored the effect of depleting KDM5B on nucleosome organization in ES cells by evaluating the distribution of DNA fragments protected from MNase digestion using average nucleosome density profiles and two-dimensional (2D) occupancy plots (see “Methods” section) [31]. While 1-dimensional (1D) occupancy plots are useful for surveying profiles of nucleosome distributions independent of fragment length, 2D plots reveal nucleosome occupancy relative to fragment length in a matrix form. Therefore, we utilized plot2DO [31] to further interrogate nucleosome occupancy profiles surrounding + 1 and − 1 nucleosomes with stratified nucleosome variance (10–50, 51–100, 101–150, 151–200 bp) between KDM5B-depleted and control ES cells (Fig. 2a, b, Additional file 1: Fig. S2). While we observed increased nucleosome enrichment nearby TSS regions for + 1 and − 1 nucleosomes that exhibit small variance (10–50 bp downstream shift) in KDM5B-depleted ES cells, nucleosome phasing was relatively synchronized between control and KDM5B-depleted ES cells (Fig. 2c, d, Additional file 1: Fig. S2C, D). However, phasing was asynchronous for + 1 and − 1 nucleosomes with moderate to high variance (51–100, 101–150, 151–200 bp downstream shift) (Fig. 2c, d, Additional file 1: Fig. S2C, D). Previous genome-wide MNase-Seq studies suggest that features of canonical nucleosome phasing include a nucleosome-depleted region around active TSS regions, a prominent + 1 nucleosome peak downstream of the TSS, and multiple phased nucleosome peaks with decaying enrichment as a function of the distance from the TSS [32]. We observed canonical nucleosome phasing around TSS regions containing + 1 or − 1 nucleosomes with small variance (downstream shift) in both KDM5B-depleted and control ES cells (Fig. 2c, d, Additional file 1: Fig. S2C, D left). However, control ES cells exhibited non-canonical phasing patterns surrounding TSS regions with moderate to high + 1 nucleosome variance (downstream shift) in KDM5B-depleted ES cells (Fig. 2c, d). In contrast, canonical phasing patterns, including a nucleosome-depleted TSS region and prominent + 1 nucleosome peaks, were largely maintained surrounding TSS regions with moderate to high + 1 nucleosome variance in KDM5B-depleted ES cells (Fig. 2c, d, middle, right). 2D plots confirm the presence of prominent + 1 nucleosome peaks for nucleosomes with high variance in KDM5B-depleted ES cells (Fig. 2d, Additional file 1: Fig. S2D).

We also evaluated nucleosome enrichment and phasing surrounding TSS regions with + 1 nucleosomes exhibiting an upstream shift (Fig. 2e, f). In this case, we observed canonical nucleosome phasing for + 1 nucleosomes with small variance (upstream shift) in control and KDM5B-depleted ES cells (Fig. 2e, f). However, KDM5B-depleted ES cells exhibited non-canonical phasing patterns surrounding TSS regions with moderate to high + 1 nucleosome variance (upstream shift). These results also demonstrate slight increases in nucleosome fragment length of + 1 and − 1 nucleosomes with high variance in KDM5B-depleted ES cells (Fig. 2d, f, Additional file 1: Fig. S2D, F). Combined, these results reveal diverse nucleosome phasing patterns in ES cells and suggest that KDM5B acts in part to regulate nucleosome phasing distributions on a genome-wide scale at distinct sets of TSSs.

**Fig. 3:**
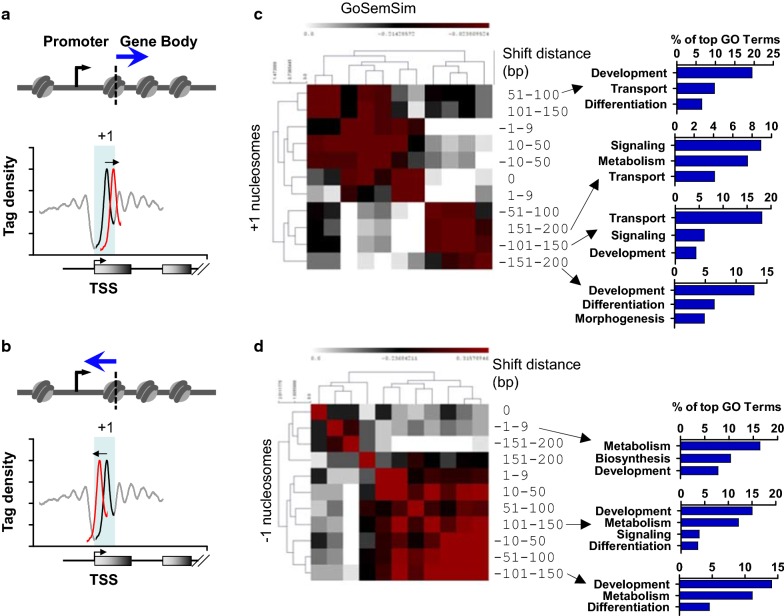
Variant nucleosomes are associated with distinct regulatory elements and genic features in KDM5B-depleted ES cells. **a**, **b** Schema of + 1 nucleosomes relative to transcriptional start site (TSS) shifted **a** downstream or **b** upstream. **c, d** Semantic similarity of GO functional annotation terms [35] enriched by GREAT [34] software for shifted **c** + 1 nucleosomes or **d** − 1 nucleosomes. Heatmaps represent semantic similarity calculated for the following sets: genes with + 1 or − 1 nucleosomes shifted by 0, 1–9, 10–51, 51–100, 101–150, or 151–200 bp in KDM5B-depleted ES cells. The three most enriched GO categories for each cluster are shown on the right. Color scale: red indicates high

Given that asynchronous and disorganized nucleosome phasing was observed at distinct sets of TSS regions in KDM5B-depleted ES cells, we evaluated the location of stable and variant + 1 and − 1 nucleosomes within genic features (Fig. 3a, b, Additional file 1: Fig. S3A, B) in KDM5B-depleted ES cells relative to control ES cells using HOMER software [33]. As expected, the majority of stable nucleosomes (0-bp shift) were localized in promoter regions (Additional file 1: Fig. S3C). However, for + 1 nucleosomes with high variance that shifted downstream in KDM5B-depleted ES cells, we observed progressively decreased enrichment of + 1 nucleosomes in promoter regions and increased enrichment of + 1 nucleosomes in intron/gene body regions (Additional file 1: Fig. S3C). In contrast, for + 1 nucleosomes with high variance that shifted upstream, we observed increased enrichment of + 1 nucleosomes in promoter regions and decreased enrichment in intron/gene body regions (Additional file 1: Fig. S3D). In contrast, − 1 nucleosomes with no variance (0-bp shift), moderate (51–100-bp shift) or high variance (101–150, 151–200-bp shift) were all enriched in promoter regions, as HOMER annotation of promoter regions includes a 1-kb region upstream of TSS (Additional file 1: Fig. S3E, F).

Furthermore, GREAT [34] functional annotation showed that variant + 1 or − 1 nucleosomes are located close to TSS regions of genes associated with development, differentiation, metabolism, and signaling. Overrepresentation of these gene ontology (GO) functional annotation terms was evident by semantic analysis of gene ontology (GO) terms [35] (Fig. 3c, d). These findings demonstrate variant + 1 and − 1 nucleosomes are associated with distinct sets of genes.

### DNA sequence features of variant nucleosomes

**Fig. 4:**
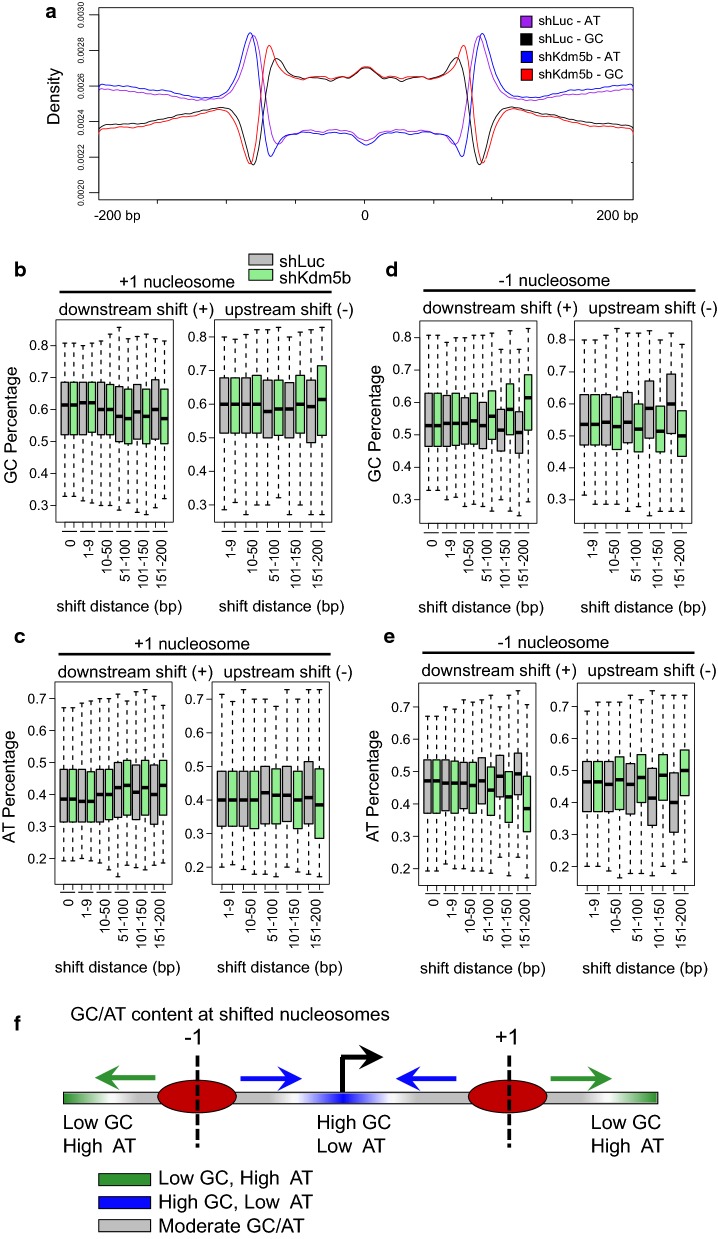
Sequence composition of regions with variant nucleosomes in KDM5B-depleted ES cells. **a** Average profile of AT and GC density at nucleosomes and flanking regions in control and KDM5B-depleted ES cells. GC sequence composition is elevated in nucleosome occupied regions relative to flanking regions, while the pattern of AT sequence composition is opposite. Variant **b**, **c** + 1 nucleosomes and **d**, **e** − 1 nucleosomes exhibit altered **b**, **d** GC and **c**, **e** AT Sequence composition in KDM5B-depleted ES cells (nucleosome shift distance: 0, 1–9, 10–50, 51–100, 101–150, 151–200 bp). **f** Schematic of nucleosome shift directionality relative to GC/AT sequence composition

Features of underlying DNA sequences are affected by nucleosome positioning [36]. In particular, nucleosomes exhibit a sequence preference for two classes of dinucleotide sequences, including AA/TT/TA/AA and GC/CG, which have variable bending properties and are positioned periodically with respect to one another in anti-phase along nucleosomal DNA [36, 37]. Global analysis of GC and AT content using the R package seqPattern showed variation in sequence preference for nucleosomes in KDM5B-depleted ES cells (Fig. 4a). Genome-wide average profiles of all nucleosomes showed increases in GC content and decreases in AT content at the leading and lagging edges of nucleosomes in KDM5B-depleted ES cells (Fig. 4a). Further analysis of GC and AT dinucleotide content at variant + 1 and − 1 nucleosomes with respect to TSSs in KDM5B-depleted ES cells showed that + 1 nucleosomes with high variance and a downstream directional shift exhibited decreased enrichment of GC dinucleotides (Fig. 4b) and increased enrichment of AT dinucleotides (Fig. 4c). In contrast, + 1 nucleosomes with high variance and an upstream directional shift displayed higher enrichment of GC dinucleotide motifs (Fig. 4b) and lower enrichment of AT sequences (Fig. 4c) in KDM5B-depleted ES cells. We also analyzed GC and AT dinucleotide enrichment at − 1 nucleosomes with high variance. In contrast to results we observed for + 1 nucleosomes with high variance (Fig. 4b, c), GC and AT content analysis revealed that − 1 nucleosomes with high variance and a downstream directional shift exhibited increased enrichment of GC dinucleotides (Fig. 4d) and decreased enrichment of AT dinucleotides (Fig. 4e). Also, − 1 nucleosomes with high variance and an upstream directional shift displayed lower enrichment of GC dinucleotide motifs (Fig. 4d) and higher enrichment of AT sequences (Fig. 4e). Overall, these findings reflect a correlation between positional changes of variant nucleosomes and the underlying sequences being proximal or distal to promoter regions (Fig. 4f).

### DNA shape features of sequences containing variant nucleosomes and altered H3K4 methylation

**Fig. 5:**
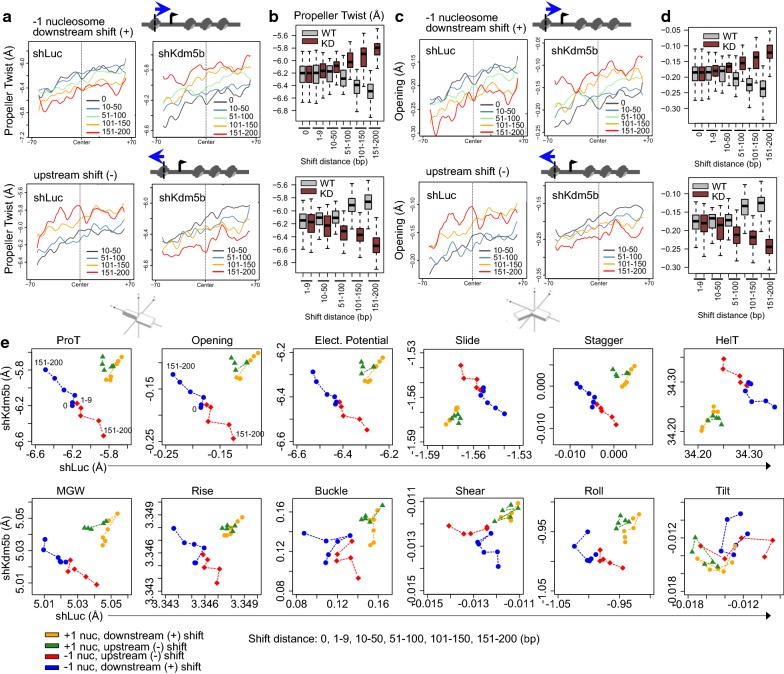
DNA shape and sequence features of variant nucleosomes in KDM5B depleted ES cells. Average profiles of DNA shape and sequence features of regions with variant **a**–**d** − 1 nucleosomes in KDM5B-depleted ES cells (nucleosome shift distance: 0, 1–9, 10–50, 51–100, 101–150, 151–200 bp). **a**, **b** Propeller twist and **c**, **d** opening, **a**, **c** average profiles and **b**, **d** boxplots of sequences with downstream (top) or upstream (bottom) shifted − 1 nucleosomes (black line, 0-bp; blue, 10–50-bp; green, 51–100-bp; orange, 101–150-bp; red, 151–200-bp shift). Note that 151–200-bp shifted nucleosomes in KDM5B-depleted ES cells exhibit altered propeller twist and opening relative to control ES cells. Schematic representation of propeller twist and opening DNA shape features are also shown. **e** Scatter plot of normalized values for 12 DNA shape and sequence features [43], including propeller twist (ProT), opening, electrostatic potential (EP), slide, stagger, helix twist (HelT), minor groove width (MGW), rise, buckle, shear, roll, tilt of variant + 1 and − 1 nucleosomes in KDM5B-depleted ES cells

DNA shape has been shown to play a critical role in protein-DNA recognition [38, 39]. Evidence also suggests that DNA methylation may influence nucleosome positioning [40, 41]. Posttranslational modification of histone tails can also alter interactions between histones and nucleosomal DNA [42]. However, it is unclear whether variant nucleosomes in KDM5B-depleted ES cells, with altered H3K4 methylation profiles, exhibit a conformational preference for free DNA. Therefore, we investigated whether DNA shape is a partial determinant of nucleosome positioning in KDM5B-depleted ES cells. To gain insight into the DNA shape basis for differences in nucleosome positioning in control and KDM5B-depleted ES cells, we evaluated the contribution of DNA shape features [43, 44]. DNA shape software [43, 44] was used to predict intrinsic DNA shapes of sequences containing nucleosomes. Most notably, variant − 1 nucleosomes exhibited a preference for sequences with altered features such as propeller twist (Fig. 5a, b, e), opening (Fig. 5c–e), electrostatic potential (Fig. 5e), minor groove width (MGW) (Fig. 5e), rise (Fig. 5e), stagger (Fig. 5e), helix twist (HelT) (Fig. 5e), and shear and roll (Fig. 5e). Variant − 1 nucleosomes that shifted downstream in KDM5B-depleted ES cells preferred sequences with increased propeller twist, opening, electrostatic potential, stagger, MGW, rise, and buckle, while − 1 variant nucleosomes that shifted upstream preferred sequences with decreased propeller twist, opening, electrostatic potential, stagger, MGW, rise, and buckle (Fig. 5e). Affinity of canonical (0-bp shift) and variant nucleosomes (50–200-bp shift) in control and KDM5B-depleted ES cells, respectively, for sequences of varying DNA shape features are shown in Fig. 5e. We also observed sequence preferences for variant + 1 nucleosomes that shifted upstream or downstream in KDM5B-depleted ES cells (Additional file 1: Figs. S4–S6). Combined, these findings suggest that DNA shape predicts sequence preferences of canonical nucleosomes and variant nucleosomes. These results also suggest that histone DNA binding patterns such as bending or electrostatic interactions may be influenced by posttranslational modifications such as H3K4 methylation. This DNA shape augmented model approach provides additional insight into mechanisms of optimal positioning of nucleosomes in control and KDM5B-depleted ES cells.

### Association of altered nucleosome positioning with changes in H3K4me3 and gene expression in KDM5B-depleted ES cells

**Fig. 6:**
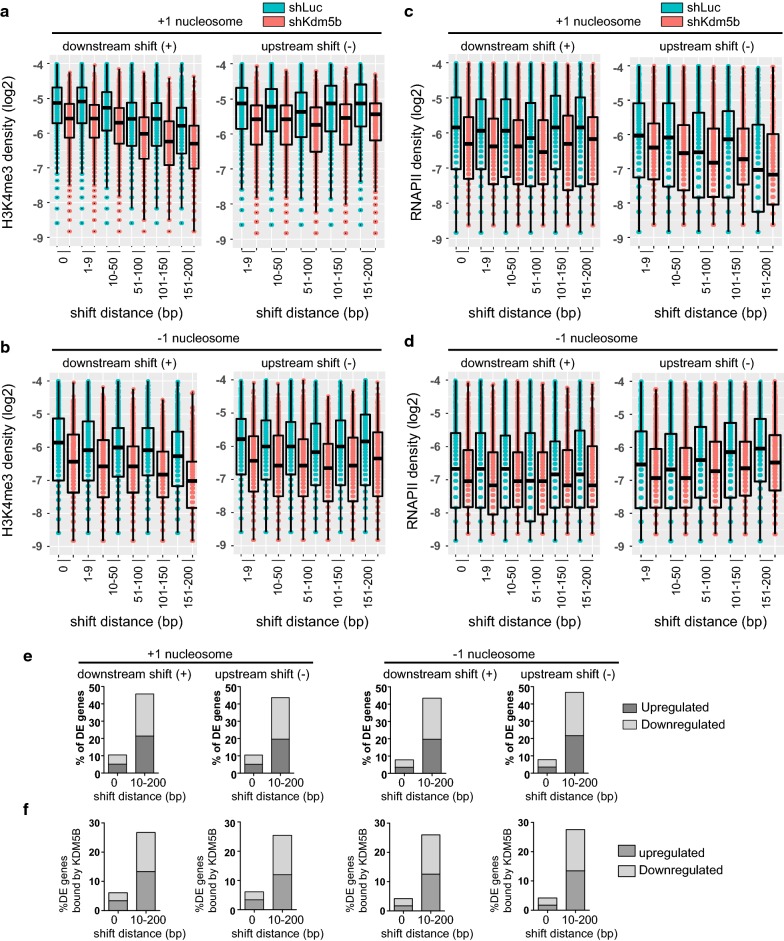
Characterization of H3K4me3, RNAPII and expression of genes with altered nucleosome distributions. **a**–**d** Boxplots of **a**, **b** H3K4me3 (log2) and **c**, **d** RNAPII (log2) densities at regions with shifted + 1 and − 1 nucleosomes relative to TSS regions in shLuc and shKdm5b ES cells. Shift distance (bp). **e** RNA-Seq expression analysis. Percentage of upregulated or downregulated differentially expressed (DE) genes with uniform nucleosome placement (0-bp shift) or shifted + 1 or − 1 nucleosomes (10–200-bp shift) relative to TSS in KDM5B-depleted ES cells. **f** Percentage of upregulated or downregulated DE genes, bound by KDM5B, with uniform or shifted nucleosome placement relative to TSS in KDM5B-depleted ES cells

Our previous findings demonstrate that KDM5B focuses H3K4 methylation nearby promoter regions of active genes to prevent the spread of H3K4 methylation from TSS to gene body regions [18]. Depletion of KDM5B resulted in decreased H3K4me3/2 at promoter regions and increased H3K4me3/2 in gene body regions. These results also demonstrated that KDM5B co-localizes with H3K4me3 at active genes and at bivalently marked (H3K4me3/H3K27me3) genes [18]. The redistribution of H3K4 methylation was also correlated with changes in gene expression [18, 24] and the transcriptional cycle including RNA polymerase II (RNAPII) occupancy in ES cells [24]. However, it is unclear whether changes in H3K4 methylation, RNAPII binding, or gene expression are correlated with changes in nucleosome positioning in KDM5B-depleted ES cells. To investigate whether alterations in nucleosome positioning are associated with changes in H3K4me3, we analyzed ChIP-Seq data [18]. Integration of H3K4me3 ChIP-Seq data with MNase-Seq data revealed a negative correlation between the level of H3K4me3 and the degree of variance for + 1 or − 1 nucleosomes that shifted downstream (Fig. 6a, b). While H3K4me3 levels decreased at stable and variant + 1 or − 1 nucleosomes in KDM5B-depleted ES cells, the overall level of H3K4me3 was higher at stable nucleosomes than variant nucleosomes. However, we did not identify a correlation between H3K4me3 level and shift distance for variant nucleosomes that shifted upstream (Fig. 6a, b). These findings suggest that nucleosomes with lower levels of H3K4me3 are more sensitive to changes in H3K4me3, which is correlated with alterations in nucleosome positioning. In contrast, regions of higher levels of H3K4me3 may be more refractory to H3K4me3 induced changes in nucleosome positioning.

We also investigated whether alterations in nucleosome positioning are associated with changes in RNAPII binding [24]. In this case, we found a negative correlation between the level of RNAPII occupancy and variance of + 1 nucleosomes that shifted upstream (Fig. 6c, d). This result could suggest that an upstream shift of + 1 nucleosomes may impede RNAPII occupancy nearby TSS regions.

To investigate a relationship between nucleosome shift distance and gene expression in control and KDM5B-depleted ES cells, we analyzed RNA-Seq data [17]. These results revealed that 3221 genes are differentially expressed between control and KDM5B-depleted ES cells (> 1.5 fold-change, FDR < 0.001). Moreover, genes associated with shifted + 1 or − 1 nucleosomes are more sensitive to transcriptional dysregulation (Fig. 6e). These findings suggest that a redistribution of nucleosomes nearby TSSs is correlated with gene expression changes in KDM5B-depleted ES cells. We also investigated a relationship between KDM5B binding [18], nucleosome shift distance, and transcriptional dysregulation in KDM5B-depleted ES cells. These findings show that depletion of KDM5B is associated with alterations in nucleosome positioning and transcriptional dysregulation (Fig. 6f).

### Nucleosome occupancy at pluripotency regulators in KDM5B-depleted ES cells

**Fig. 7:**
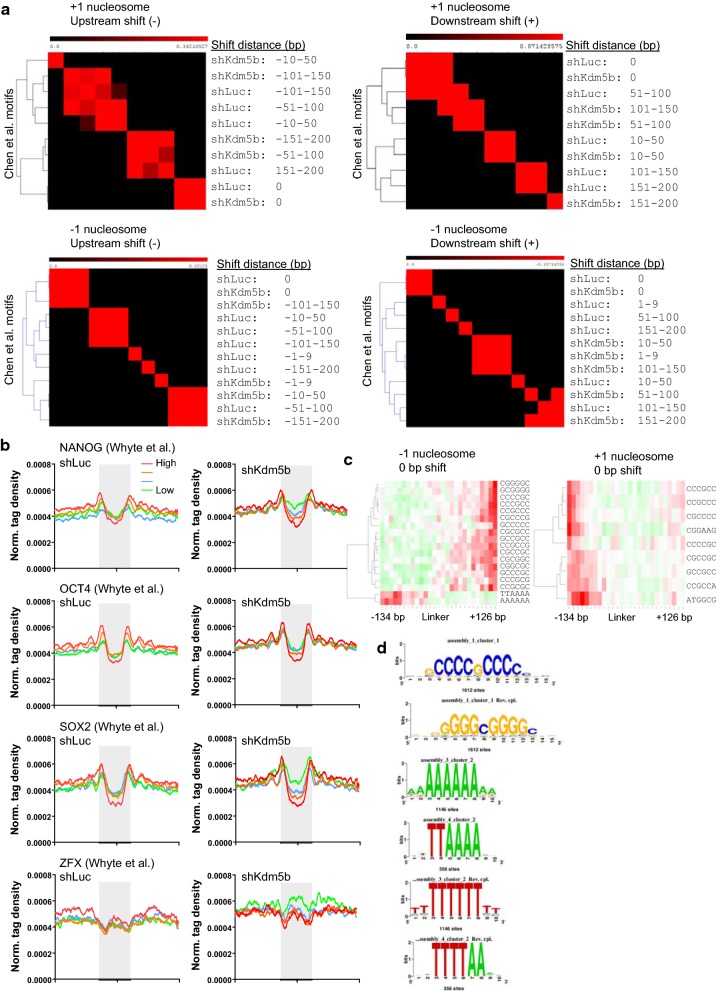
Sequence motifs enriched in regions of variant nucleosomes in KDM5B-depleted ES cells. **a** Hierarchical clustering of transcription factor binding site motifs in linker regions of shifted nucleosomes relative to the TSS (+ 1 nucleosome, upstream shift; + 1 nucleosome, downstream shift; − 1 nucleosome, upstream shift; − 1 nucleosome, downstream shift) in KDM5B-depleted ES cells. The color scale indicates enrichment (red) or underrepresentation (black) of motif occurrences. AutoSOME [68] was used to generate pair-wise affinity values. **b** Average MNase-Seq profiles around NANOG [69], OCT4, SOX2, and ZFX transcription factor binding sites in shLuc (left panel) and shKdm5b ES cells (right panel; red, highest ChIP-Seq signal; green, lowest ChIP-Seq signal). **c** Hierarchical clustering heatmap of oligonucleotide occurrences at shifted nucleosomes relative to the TSS (+ 1 nucleosome, and − 1 nucleosome) in KDM5B-depleted ES cells. The positional profiles of 6-mer motifs in 10-bp windows 130 bp upstream and downstream of the nucleosome summit are represented in the heatmap. Each row represents one *k*-mer. Color scale: the normalized frequency of *k*-mers corresponds to over- or underrepresentation. **d** MEME-ChIP confirmation of GC and AT motifs at sequences of shifted nucleosomes. Representative sequence logos of GC- and AT-rich motifs are shown

Transcription factors (TF) important for ES cell pluripotency such as OCT4, NANOG, and SOX2 have been shown to occupy nucleosome-depleted regions [45, 46], and TF binding to chromatin may be influenced by nucleosome positioning or vice versa. Because phasing of nucleosomes around regions flanking TF binding sites has been observed previously [47, 48], evaluation of TF binding may provide insight into a relationship between nucleosome positioning and TF binding to local chromatin sequences. Using MNase-Seq nucleosome positioning data, we investigated the presence of pluripotency regulator factor motifs [29] in linker regions of stable (0-bp shift) and variant nucleosomes in control and KDM5B-depleted ES cells using MEME-ChIP [49]. Hierarchical clustering of motif enrichment scores revealed differential enrichment of pluripotency TF binding motifs in sequences nearby stable nucleosomes relative to variant nucleosomes (Fig. 7a). Moreover, highly variant nucleosomes clustered closer together than stable nucleosomes or nucleosomes with low variance. Our analyses also reveal differences in phasing patterns of TF binding around nucleosomes in control and KDM5B-depleted ES cells (Fig. 7b). In particular, we observed variation in NANOG and ZFX patterns around nucleosomes in KDM5B-depleted ES cells (Fig. 7b). We extended our analyses by evaluating the distribution of oligonucleotides (“*k*-mer”) around nucleosomes. Positional analysis was performed using RSAT [50, 51] to count *k*-mers 125 bp upstream and downstream of the nucleosome summit. These results revealed a positional bias of GC-rich and AT-rich sequences in regions around − 1 and + 1 nucleosomes (Fig. 7c). MEME-ChIP [49] confirmed a positional bias of GC-rich and AT-rich sequences around − 1 and + 1 nucleosomes (Fig. 7d). Altogether, these results implicate a role for KDM5B in regulating nucleosome positioning in ES cells.

## Discussion

In this study, we utilized high-resolution MNase-Seq to investigate the role for the H3K4 demethylase, KDM5B, in regulating nucleosome positioning on a global scale in ES cells. While H3K4me3 [6] and KDM5B [18] are enriched at TSSs of active genes, and nucleosomes exhibit phasing relative to TSS regions, the role for KDM5B in regulating nucleosome positioning is largely unknown. Our results presented in this study demonstrate that KDM5B is a critical regulator of nucleosome positioning in ES cells. Depletion of KDM5B resulted in a redistribution of nucleosomes around regulatory regions including promoters, and asynchronous nucleosome phasing. Variation in nucleosome positioning is context specific, where positioning depends on genomic sequence and gene regulatory features such as promoters. Features of DNA shape also contribute to differences in nucleosome positioning in KDM5B-depleted ES cells. Variant nucleosomes in KDM5B-depleted ES cells may have a preference for sequences with specific conformational properties. Context-specific preferences may be correlated with H3K4 methylation level, position relative to gene regulatory regions, gene expression level, or the transcriptional cycle. Along this line, our results show that sequences nearby variant nucleosomes and gene regulatory regions such as promoters exhibit specific conformational properties.

Our results also show that active genes have a nucleosome-depleted region (NDR) upstream of the TSS and generally lack nucleosomes between the − 1 and + 1 nucleosomes. Promoter regions may be occupied by a RNAPII complex or chromatin remodeling complex, which has been suggested previously [31]. We also observed correlations between altered H3K4me3 levels, gene expression, and nucleosome variation. These nucleosome maps provide a useful model to investigate correlations between nucleosome positioning, H3K4 methylation, and gene expression, and for understanding heterogeneity in nucleosome spacing across different sets of genes.

There are several potential mechanisms for how KDM5B may influence nucleosome positioning. First, alterations in H3K4 methylation due to depletion of KDM5B may lead to altered accessibility. While hyperacetylation of histone tails results in modest increases in chromatin accessibility [52, 53], it is unclear whether H3K4 methylation affects nucleosome positioning in vivo directly. Changes in H3K4 methylation levels in KDM5B-depleted cells may lead to altered interactions between nucleosomes and chromatin remodeling enzymes, which position nucleosomes along DNA. Along this line, several chromatin remodelers such as SMARCA5, SMARCA1, the NuRD complex protein, SIN3A, CHD1, and CHD8 were found to interact with H3K4me3 [54]. Chromatin remodelers may cooperate with other chromatin constituents to read histone posttranslational modifications (PTMs), such as H3K4 methylation, and remodel chromatin and nucleosome placement accordingly. In this case, spreading of H3K4 methylation from promoter to gene body regions in KDM5B-depleted ES cells may direct chromatin remodelers to pattern nucleosome placement along an asynchronous array relative to normal positioning. It is important to note that we did not observe differential expression of SWI/SNF ATP-dependent chromatin remodelers such as BRG1/SMARCA4 in KDM5B-depleted ES cells. Second, KDM5B may interact with ATP-dependent chromatin remodeling factors [55]. In support of this possibility, KDM5A and KDM5B have been shown to interact with the NuRD complex in cancer cells [56, 57]. Third, altered transcriptional dynamics in KDM5B-depleted ES cells may lead to dysregulated nucleosome positioning. Decreased RNAPII occupancy at promoters regions in KDM5B-depleted ES cells [24], which are generally depleted of nucleosomes at active genes, may influence nucleosome positioning. Moreover, altered RNAPII initiation and elongation rates in KDM5B-depleted ES cells may affect nucleosome positioning. Nucleosomes can inhibit the rate of transcription by occupying regulatory sequences, and nucleosomes represent a barrier to RNAPII transcription [58]. We posit that in this kinetic model, the rate of RNAPII promoter clearance may affect nucleosome positioning. Fourth, dynamic interactions of KDM5B with chromatin constituents may affect nucleosome positioning. In this case, loss of KDM5B binding to chromatin may lead to altered nucleosome positioning. Also, our results demonstrate that expression of other KDM5 family members was unaltered in KDM5B-depleted ES cells relative to control ES cells (Additional file 1: Fig. S1), thus arguing against the possibility that altered expression of other KDM5 family members may lead to changes in H3K4 methylation and nucleosome positioning. These scenarios present intriguing possibilities for regulation of nucleosome positioning by KDM5B.

Previously, we found that depletion of KDM5B leads to delayed ES cell differentiation and dysregulated gene expression [17], which is in alignment with results from several studies [15, 19, 59]. There are several possible explanations for how alterations in nucleosome positioning in KDM5B-depleted ES cells may influence differentiation. Dysregulation of nucleosome positioning in KDM5B-depleted ES cells may contribute to dysregulated gene expression, nucleosome turnover, and affect response of chromatin to environmental stimuli such as intrinsic and external signals, all of which may contribute to delayed differentiation of KDM5B-depleted ES cells.

Moreover, it is possible that other KDM5 family members may play a role in regulating nucleosome positioning in ES cells or other cells. Results from previous studies suggest that KDM5B and other KDM5 family members may perform partially overlapping functions in ES cells [14]. Along this line, KDM5A was found to regulate ES cell differentiation [60], and KDM5C was shown to bind promoter and enhancer regions in ES cells [61]. However, because depletion of KDM5A or KDM5B impairs ES cell differentiation, and H3K4me3 levels increase in KDM5A or KDM5B-depleted ES cells, it is likely that KDM5 enzymes perform distinct functions.

## Conclusions

In summary, our results describe a novel function of KDM5B in regulating nucleosome positioning in ES cells, where KDM5B-depletion leads to a redistribution of nucleosomes nearby TSSs of active genes in ES cells. These findings also serve as a resource for modeling associations between changes in H3K4 methylation and nucleosome positioning on a global scale.

## Methods

### ES cell culture

Mouse R1 ES cells were cultured on irradiated MEFs in DMEM/15% FBS media containing LIF (ESGRO) and 1 µg/mL puromycin at 37 °C with 5% CO_2_. shLuc and shKdm5b (R1) ES cells were generated and cultured as previously described with minor modifications [17, 18, 24]. Briefly, mouse R1 ES cells were transduced with lentiviral particles encoding shRNA sequences. Twenty-four hours post-transduction, KDM5B knockdown (shKdm5b) ES cells and control Luciferase-shRNA (shLuc) ES cells were stably selected in the presence of puromycin for 48–72 h, expanded for several days (3–4 days), and subsequently harvested for MNase-Seq experiments.

For MNase-Seq experiments, ES cells were cultured on gelatin-coated dishes in ES cell media containing 1.5 µM CHIR9901 (GSK3 inhibitor) for several passages to remove feeder cells. ES cells were passed by washing with PBS, and dissociating with trypsin using serological pipettes (sc-200279, sc-200281).

### MNase-Seq analysis

MNase-Seq experiments were performed as described previously with modifications [62]. Briefly, 15e6 mouse ES cells (R1) were harvested and chemically crosslinked with 1% formaldehyde (Sigma) for 5–10 min at 37 °C. Crosslinking was quenched by addition of 1.25 M glycine to a final concentration of 0.125 M, washed with PBS twice, and the pellet was flash frozen in liquid nitrogen. Cells were thawed on ice in PBS + 0.5% Triton X-100 (lyse buffer), and nuclei were pelleted by centrifugation at 350 g for 5 min at 4 °C. Nuclei were washed with 1 mL MNase digestion buffer (10 mM tris-HCl (pH7.4), 15 mM NaCl, 60 mM KCl, 0.15 mM spermine, 0.5 mM spermidine), centrifuged for 5 min at 4 °C, and re-suspended in 800 µL MNase digestion buffer. The concentration of Ca^2+^ was adjusted to 1 mM with 1 M CaCl_2_. The nuclei suspension was aliquoted into tubes containing 100 µL of serially diluted MNase (0.5 U, 0.25 U, 0.125 U, 0.06 U; 1:2–1:16 dilution). The reaction was incubated for 5 min at 37 °C, and subsequently stopped by addition of stop buffer (20 mM EDTA, 20 mM EGTA, 0.4% SDS, 0.5 mg/mL proteinase K). Next, the samples were incubated at 65 °C overnight, and DNA was extracted using phenol/chloroform and precipitated with ethanol in the presence of Glycoblue. MNase-enriched DNA was run on a 2% agarose gel, and mononucleosome-sized DNA fragments were cut and pooled; MNase-enriched DNA was subjected to end-repair using an End-It DNA End-Repair kit (Epicentre), followed by addition of a single A nucleotide, and ligation of custom Illumina adapters. PCR was performed using Phusion High Fidelity PCR master mix. MNase libraries were sequenced on Illumina HiSeq platforms according to the manufacture’s protocol. Paired-end MNase-Seq reads were mapped to the mouse genome (mm9) using bowtie2 [63] with default settings, and redundant reads were removed from further analysis. At least two replicates were performed for the MNase-Seq analyses.

MNase-Seq read enriched regions (peaks) were further investigated using Danpos, which was used to evaluate nucleosome position shifts and occupancy changes using a uniform statistical framework [30]. The RPBM measure (read per base per million reads) was used to quantify the density at genomic regions from MNase-Seq datasets, and the UCSC genome browser was used to visualize this data.

### Nucleosome occupancy relative to TSS, DNase I hypersensitive sites, CTCF-bound regions from MNase-Seq data

MNase-Seq tag densities were calculated around TSS (Fig. 1a), DHS regions (Fig. 1b), CTCF binding sites (Fig. 1c) by normalizing tag counts to total mapped tag counts. Average profiles were subsequently plotted. The positional distributions of shLuc (control) MNase-Seq reads relative to TSS, DNase I hypersensitive sites (DHS), and CTCF are consistent with previous reports [3, 27, 37, 64].

### Nucleosome fragment length

Nucleosome fragments (Fig. 1d) were identified from paired-end shLuc and shKdm5b MNase-Seq data using publically available software (https://github.com/binbinlai2012/scMNase) [3]. Nucleosome fragments with a length of 140–180 bp were considered canonical nucleosomes.

### Nucleosome variance

Nucleosome variance is defined as the distance between two overlapping nucleosomes [3] across shLuc and shKdm5b ES cells. Nucleosome variance was calculated using publically available software (https://github.com/binbinlai2012/scMNase). Briefly, nucleosome variance was measured by averaging all variances between nucleosome pairs within regions.

### Nucleosome spacing uniformness

Uniformness of nucleosome spacing was calculated using publically available software (https://github.com/binbinlai2012/scMNase).

### 2D nucleosome occupancy heatmaps

2D nucleosome occupancy heatmaps were generated using the R package plot2DO [31].

### ChIP-Seq analysis

H3K4me3 [18] and RNAPII [24] ChIP-Seq data were mapped to the mouse genome (mm9) using bowtie2 [63] with default settings, and redundant reads were removed from further analysis. ChIP-Seq read enriched regions (peaks) were identified relative to control Input DNA using “Spatial Clustering for Identification of ChIP-Enriched Regions” (SICER) software [65] with a window size setting of 200 bps, a gap setting of 400 bps, and a FDR setting of 0.001. The RPBM measure (read per base per million reads) was used to quantify the density at genomic regions from ChIP-Seq datasets. Kolmogorov–Smirnov tests were used to obtain *p*-value statistics and compare densities at genomic regions.

### RNA-Seq analysis

RNA-Seq data [17] was mapped to the mouse genome (mm9) using bowtie2 [63] with default settings. The RPKM measure (read per kilobases of exon model per million reads) [66] was used to quantify the mRNA expression level of a gene from RNA-Seq data. Differentially expressed genes were identified using edgeR [false discovery rate (FDR) < 0.001; fold-change (FC) > 1.5] [67].

### Q-RT-PCR

RNA extraction and quantitative real-time PCR (Q-RT-PCR) were performed as previously described with minor modifications [17]. Total RNA was harvested from ES cells using a Qiagen RNeasy Mini Kit and DNase treated on-column. Reverse transcription was performed using an Invitrogen Superscript III kit. Primers were designed using the Roche Universal Probe Library Assay design Center.

## Additional file

**Additional file 1: Figure S1.** Expression of KDM5 family members in KDM5B-depleted ES cells. (**A**) Quantitative real-time Q-RT-PCR expression analysis of KDM5 family members in control and KDM5B-depleted ES cells. **Figure S2.** Characterization of altered nucleosome distributions in KDM5B depleted ES cells. (**A**–**B**) Schema of −1 nucleosomes relative to transcriptional start sites (TSS) shifted (**A**) downstream or (**B**) upstream. (**C**) Average profiles of nucleosomes around TSS of genes with −1 nucleosomes shifted downstream in KDM5B-depleted ES cells (shift distance: 0, 1–9, 10–50, 51–100, 101–150, 151–200 bp). Note the altered nucleosome densities and asynchronous array of nucleosomes in KDM5B-depleted ES cells relative to control ES cells. (**D**) Two-dimensional (2D) occupancy plot of nucleosomes around TSS of genes with −1 nucleosomes shifted upstream in KDM5B-depleted ES cells. Plot2DO(31) was used to show relative nucleosome occupancy and fragment length in a heatmap matrix. Average profile of nucleosome occupancy is shown above the heatmap plot. X-axis: position relative to center of nucleosome; y-axis: fragment length (bp). (**E**) Average profiles (relative to TSS) of −1 nucleosomes shifted upstream. (**F**) Two-dimensional (2D) occupancy plot of nucleosomes around TSS of genes with −1 nucleosomes shifted upstream in KDM5B-depleted ES cells. **Figure S3.** Variant nucleosomes are associated with distinct regulatory elements and genic features in the KDM5B depleted ES cells. (**A**–**B**) Schema of −1 nucleosomes relative to transcriptional start site (TSS) shifted (**A**) downstream or (**B**) upstream. (**C**–**F**) HOMER (40) functional annotation of regions enriched with (**C**, **E**) downstream or (**D**, **F**) upstream shifted nucleosomes in KDM5B-depleted ES cells. **Figure S4.** DNA shape and sequence features of variant nucleosomes in KDM5B depleted ES cells. Average profiles of DNA shape and sequence features of regions with variant (**A**–**D**) +1 nucleosomes in KDM5B-depleted ES cells (nucleosome shift distance: 0, 1–9, 10–50, 51–100, 101–150, 151–200 bp). (**A**–**B**) Propeller Twist and (C-D) Opening (**A**, **C**) average profiles and (**B**, **D**) boxplots of sequences with downstream (top) or upstream (bottom) shifted +1 nucleosomes (black line, 0 bp; blue, 10–50 bp; green, 51–100 bp; orange, 101–150 bp; red, 151–200 bp shift). Note that 151–200 bp shifted nucleosomes in KDM5B-depleted ES cells exhibit altered Propeller Twist and Opening relative to control ES cells. Schematic representations of Propeller Twist and Opening DNA shape features are also shown(49). **Figure S5.** Electrostatic potential and slide DNA shape and sequence features of variant nucleosomes in KDM5B depleted ES cells. Average profiles of DNA shape and sequence features of regions with variant +1 or −1 nucleosomes in KDM5B-depleted ES cells (nucleosome shift distance: 0, 1–9, 10-50, 51–100, 101–150, 151–200 bp). (**A**–**D**) Electrostastic potential (EP) and (**E**–**H**) slide (**A**, **C**, **E**, **G**) average profiles and (**B**, **D**, **F**, **H**) boxplots of sequences with downstream (top) or upstream (bottom) shifted +1 or −1 nucleosomes (black line, 0 bp; blue, 10–50 bp; green, 51–100 bp; orange, 101–150 bp; red, 151–200 bp shift). Note that 151–200 bp shifted nucleosomes in KDM5B-depleted ES cells exhibit altered electrostatic potential and slide relative to control ES cells. **Figure S6.** Stagger and helix twist DNA shape and sequence features of variant nucleosomes in KDM5B depleted ES cells. Average profiles of DNA shape and sequence features of regions with variant +1 or −1 nucleosomes in KDM5B-depleted ES cells (nucleosome shift distance: 0, 1–9, 10–50, 51–100, 101–150, 151–200 bp). (**A**–**D**) Stagger and (**E**–**H**) helix twist (**A**, **C**, **E**, **G**) average profiles and (**B**, **D**, **F**, **H**) boxplots of sequences with downstream (top) or upstream (bottom) shifted +1 or −1 nucleosomes (black line, 0 bp; blue, 10–50 bp; green, 51–100 bp; orange, 101–150 bp; red, 151–200 bp shift). Note that 151–200 bp shifted nucleosomes in KDM5B-depleted ES cells exhibit altered stagger and helix twist relative to control ES cells.
