# Supplementary material for: Contribution of H3K4 demethylase KDM5B to nucleosome organization in embryonic stem cells revealed by micrococcal nuclease sequencing
Source: Epigenetics Chromatin. 2019 Apr 2;12:20. doi: 10.1186/s13072-019-0266-9 (PMC6444878; doi:10.1186/s13072-019-0266-9)
Supplement: Supplementary file 1 — Additional file 1: Figure S1. Expression of KDM5 family members in KDM5B-depleted ES cells. (A) Quantitative real-time Q-RT-PCR expression analysis of KDM5 family members in control and KDM5B-depleted ES cells. Figure S2. Characterization of altered nucleosome distributions in KDM5B depleted ES cells. (A–B) Schema of −1 nucleosomes relative to transcriptional start sites (TSS) shifted (A) downstream or (B) upstream. (C) Average profiles of nucleosomes around TSS of genes with −1 nucleosomes shifted downstream in KDM5B-depleted ES cells (shift distance: 0, 1–9, 10–50, 51–100, 101–150, 151–200 bp). Note the altered nucleosome densities and asynchronous array of nucleosomes in KDM5B-depleted ES cells relative to control ES cells. (D) Two-dimensional (2D) occupancy plot of nucleosomes around TSS of genes with −1 nucleosomes shifted upstream in KDM5B-depleted ES cells. Plot2DO(31) was used to show relative nucleosome occupancy and fragment length in a heatmap matrix. Average profile of nucleosome occupancy is shown above the heatmap plot. X-axis: position relative to center of nucleosome; y-axis: fragment length (bp). (E) Average profiles (relative to TSS) of −1 nucleosomes shifted upstream. (F) Two-dimensional (2D) occupancy plot of nucleosomes around TSS of genes with −1 nucleosomes shifted upstream in KDM5B-depleted ES cells. Figure S3. Variant nucleosomes are associated with distinct regulatory elements and genic features in the KDM5B depleted ES cells. (A–B) Schema of −1 nucleosomes relative to transcriptional start site (TSS) shifted (A) downstream or (B) upstream. (C–F) HOMER (40) functional annotation of regions enriched with (C, E) downstream or (D, F) upstream shifted nucleosomes in KDM5B-depleted ES cells. Figure S4. DNA shape and sequence features of variant nucleosomes in KDM5B depleted ES cells. Average profiles of DNA shape and sequence features of regions with variant (A–D) +1 nucleosomes in KDM5B-depleted ES cells (nucleosome shift distance: 0, 1–9, 10–5 [file 13072_2019_266_MOESM1_ESM.pdf]

SUPPLEMENTAL MATERIAL

Figure S1. Expression of KDM5 family members in KDM5B-depleted ES cells.

(A) Quantitative real-time Q-RT-PCR expression analysis of KDM5 family members in control and KDM5B-depleted ES cells.

Figure S2. Characterization of altered nucleosome distributions in KDM5B depleted ES cells

(**A-B**) Schema of -1 nucleosomes relative to transcriptional start sites (TSS) shifted (**A**) downstream or (**B**) upstream. (**C**) Average profiles of nucleosomes around TSS of genes with -1 nucleosomes shifted downstream in KDM5B-depleted ES cells (shift distance: 0, 1-9, 10-50, 51-100, 101-150, 151-200 bp). Note the altered nucleosome densities and asynchronous array of nucleosomes in KDM5B-depleted ES cells relative to control ES cells. (**D**) Two-dimensional (2D) occupancy plot of nucleosomes around TSS of genes with -1 nucleosomes shifted upstream in KDM5B-depleted ES cells. Plot2DO(31) was used to show relative nucleosome occupancy and fragment length in a heatmap matrix. Average profile of nucleosome occupancy is shown above the heatmap plot. X-axis: position relative to center of nucleosome; y-axis: fragment length (bp). (**E**) Average profiles (relative to TSS) of -1 nucleosomes shifted upstream. (**F**) Two-dimensional (2D) occupancy plot of nucleosomes around TSS of genes with -1 nucleosomes shifted upstream in KDM5B-depleted ES cells.

Figure S3. Variant nucleosomes are associated with distinct regulatory elements and genic features in the KDM5B depleted ES cells

(A-B) Schema of -1 nucleosomes relative to transcriptional start site (TSS) shifted (**A**) downstream or (**B**) upstream. (**C-F**) HOMER (40) functional annotation of regions enriched with (**C, E**) downstream or (**D, F**) upstream shifted nucleosomes in KDM5B-depleted ES cells.

Figure S4. DNA shape and sequence features of variant nucleosomes in KDM5B depleted ES cells

Average profiles of DNA shape and sequence features of regions with variant (**A-D**) +1 nucleosomes in KDM5B-depleted ES cells (nucleosome shift distance: 0, 1-9, 10-50, 51-100, 101-150, 151-200 bp). (**A-B**) Propeller Twist and (**C-D**) Opening (**A, C**) average profiles and (**B, D**) boxplots of sequences with downstream (top) or upstream (bottom) shifted +1 nucleosomes (black line, 0 bp; blue, 10-50 bp; green, 51-100 bp; orange, 101-150 bp; red, 151-200 bp shift). Note that 151-200 bp shifted nucleosomes in KDM5B-depleted ES cells exhibit altered Propeller Twist and Opening relative to control ES cells. Schematic representations of Propeller Twist and Opening DNA shape features are also shown(49).

Figure S5. Electrostatic potential and slide DNA shape and sequence features of variant nucleosomes in KDM5B depleted ES cells

Average profiles of DNA shape and sequence features of regions with variant +1 or -1 nucleosomes in KDM5B-depleted ES cells (nucleosome shift distance: 0, 1-9, 10-50, 51-100, 101-150, 151-200 bp). (**A-D**) Electrostastic potential (EP) and (**E-H**) slide (**A, C, E, G**) average profiles and (**B, D, F, H**) boxplots of sequences with downstream (top) or upstream (bottom) shifted +1 or -1 nucleosomes (black line, 0 bp; blue, 10-50 bp; green, 51-100 bp; orange, 101-150 bp; red, 151-200 bp shift). Note that 151-200 bp shifted nucleosomes in KDM5B-depleted ES cells exhibit altered electrostatic potential and slide relative to control ES cells.

Figure S6. Stagger and helix twist DNA shape and sequence features of variant nucleosomes in KDM5B depleted ES cells

Average profiles of DNA shape and sequence features of regions with variant +1 or -1 nucleosomes in KDM5B-depleted ES cells (nucleosome shift distance: 0, 1-9, 10-50, 51-100, 101-150, 151-200 bp). (**A-D**) Stagger and (**E-H**) helix twist (**A, C, E, G**) average profiles and (**B, D, F, H**) boxplots of sequences with downstream (top) or upstream (bottom) shifted +1 or -1 nucleosomes (black line, 0 bp; blue, 10-50 bp; green, 51-100 bp; orange, 101-150 bp; red, 151-200 bp shift). Note that 151-200 bp shifted nucleosomes in KDM5B-depleted ES cells exhibit altered stagger and helix twist relative to control ES cells.

A

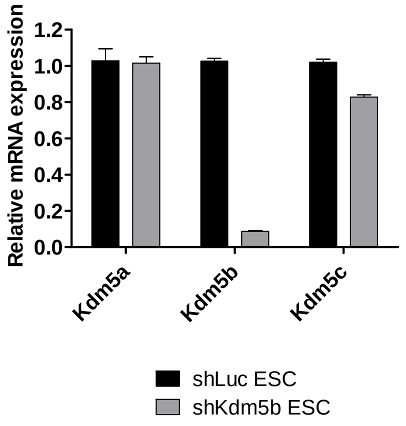
Relative mRNA expression
1.2
1.0
0.8
0.6
0.4
0.2
0.0
Kdm5a
Kdm5b
Kdm5c
shLuc ESC
shKdm5b ESC

Figure S1

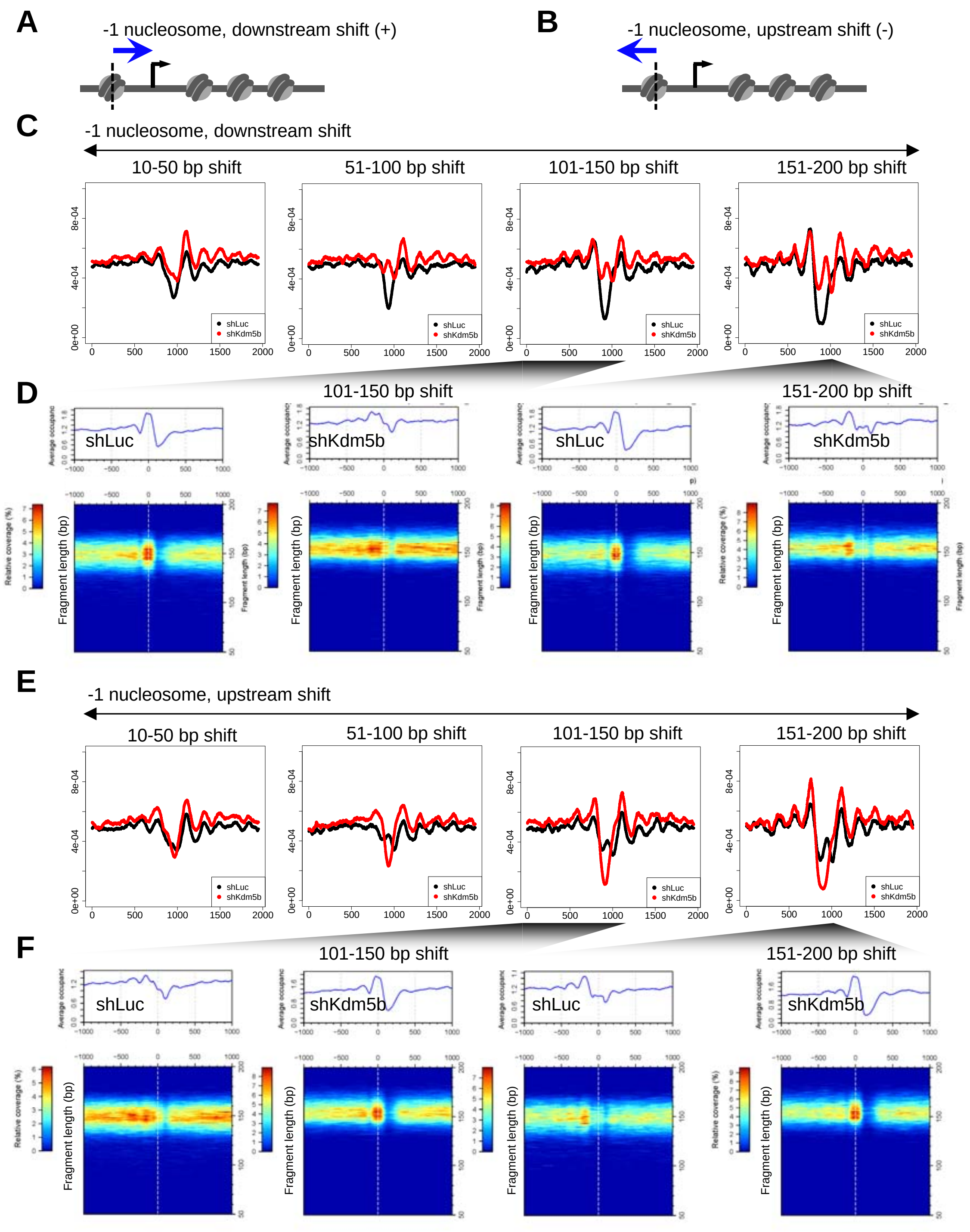
A
-1 nucleosome, downstream shift (+)
B
-1 nucleosome, upstream shift (-)
C
-1 nucleosome, downstream shift
10-50 bp shift
51-100 bp shift
101-150 bp shift
151-200 bp shift
shLuc
shKdm5b
D
101-150 bp shift
151-200 bp shift
shLuc
shKdm5b
Fragment length (bp)
E
-1 nucleosome, upstream shift
10-50 bp shift
51-100 bp shift
101-150 bp shift
151-200 bp shift
F
101-150 bp shift
151-200 bp shift
shLuc
shKdm5b
Fragment length (bp)

Figure S2

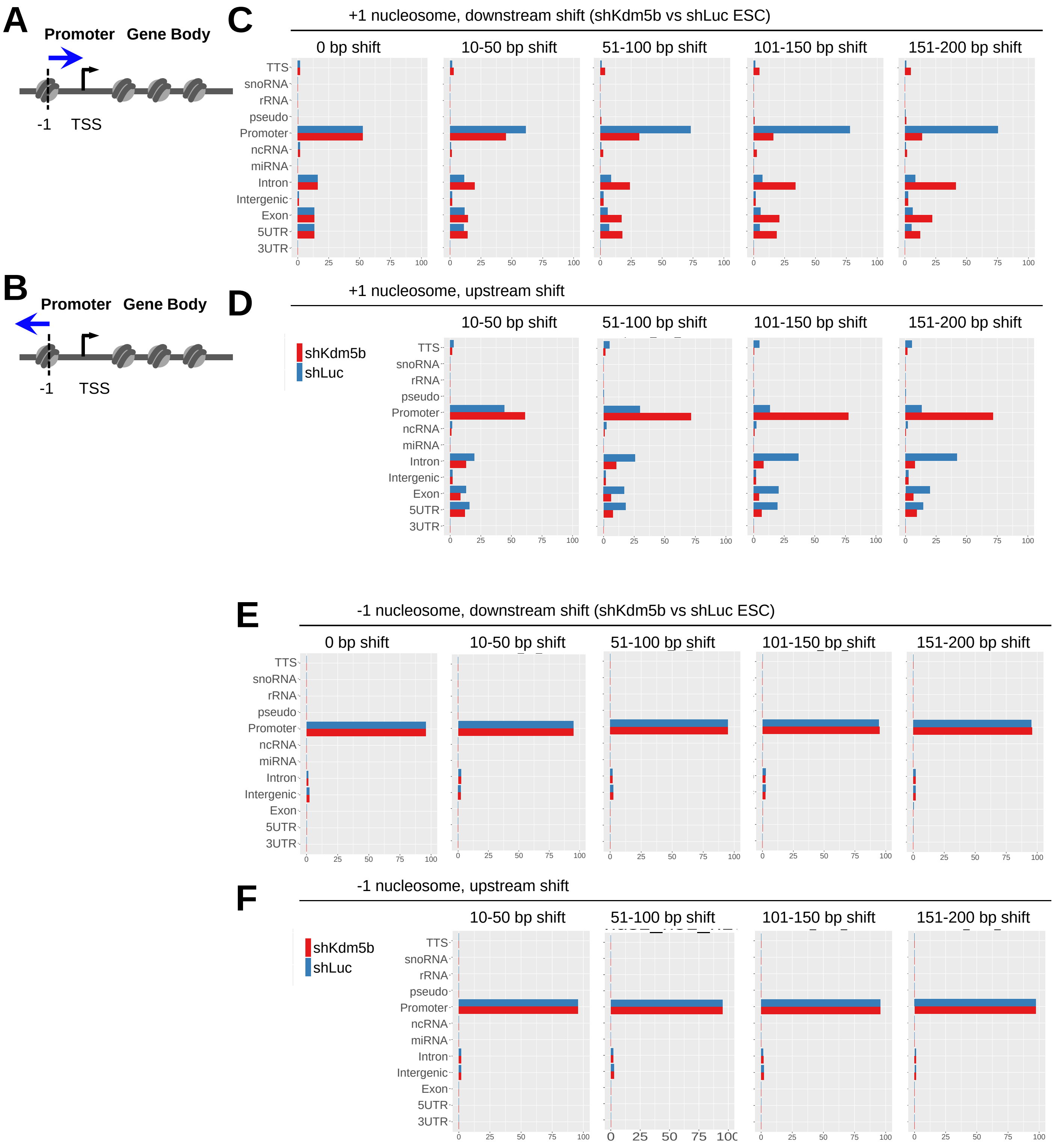
A
Promoter
Gene Body
-1
TSS
B
Promoter
Gene Body
-1
TSS
C
+1 nucleosome, downstream shift (shKdm5b vs shLuc ESC)
0 bp shift
10-50 bp shift
51-100 bp shift
101-150 bp shift
151-200 bp shift
TTS
snoRNA
rRNA
pseudo
Promoter
ncRNA
miRNA
Intron
Intergenic
Exon
5UTR
3UTR
0 25 50 75 100
D
+1 nucleosome, upstream shift
shKdm5b
shLuc
E
-1 nucleosome, downstream shift (shKdm5b vs shLuc ESC)
F
-1 nucleosome, upstream shift

Figure S3

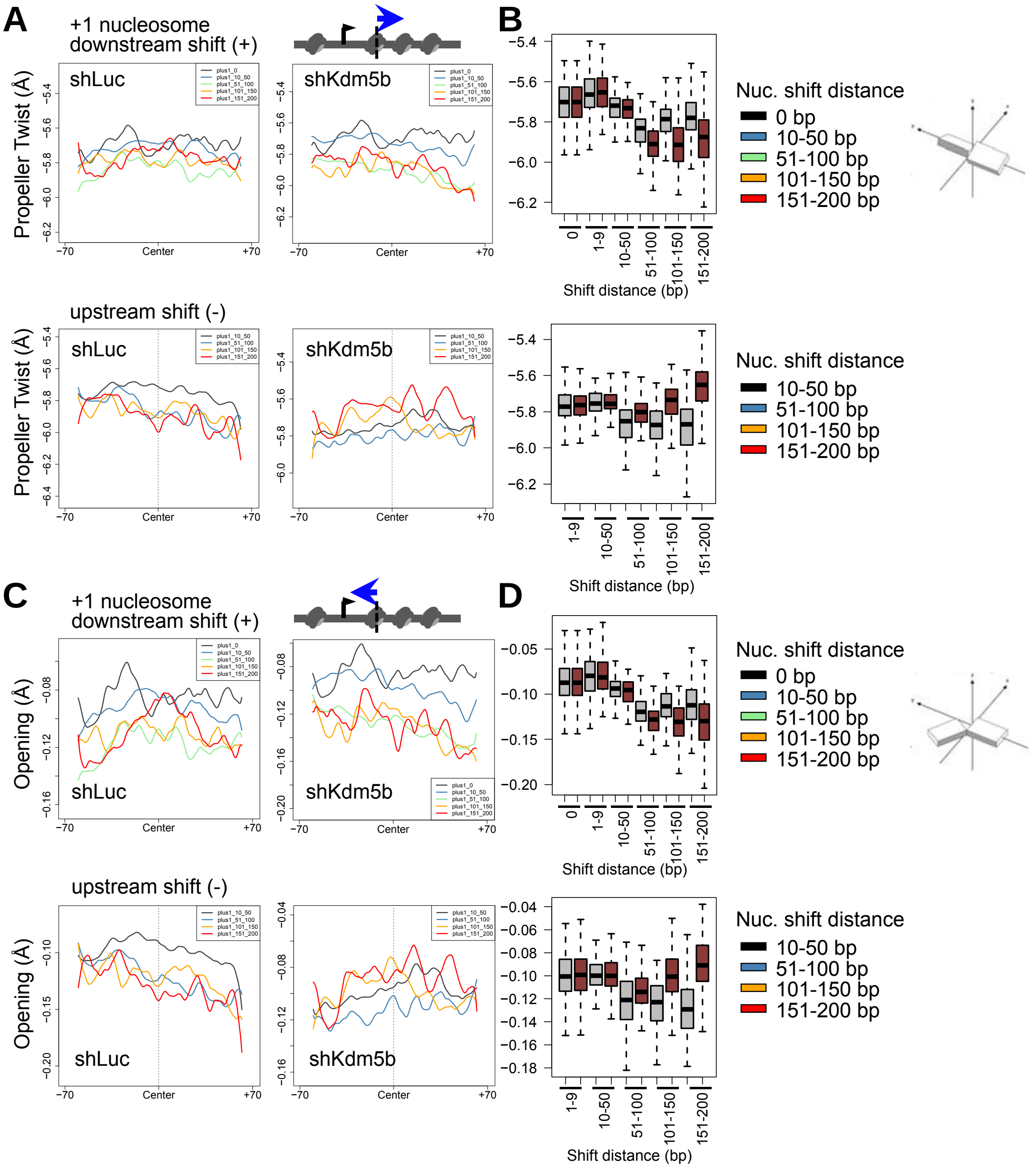
A
+1 nucleosome
downstream shift (+)
shLuc
shKdm5b
Propeller Twist (Å)
upstream shift (-)
B
Shift distance (bp)
Nuc. shift distance
0 bp
10-50 bp
51-100 bp
101-150 bp
151-200 bp
C
Opening (Å)
D

Figure S4

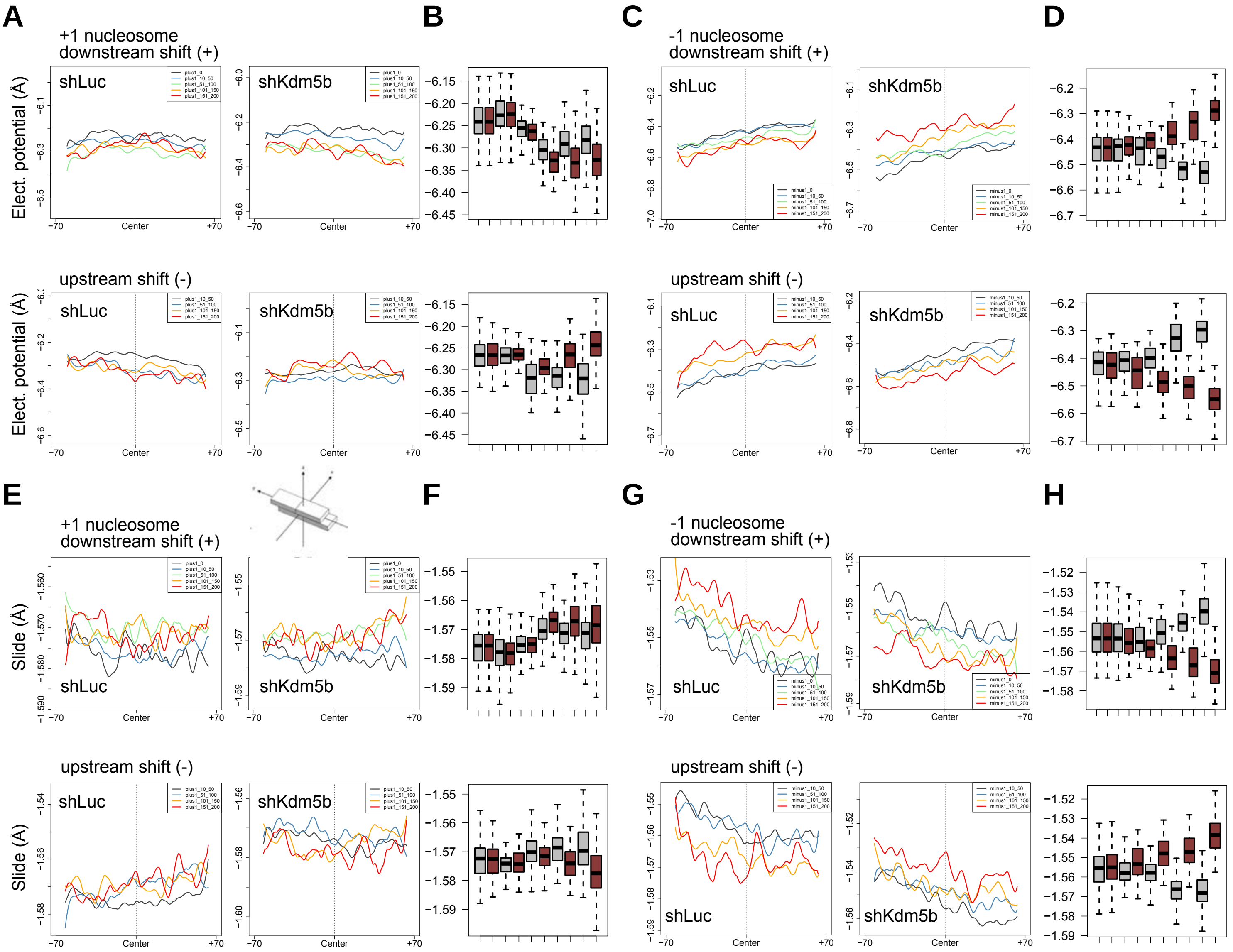
A
+1 nucleosome
downstream shift (+)
shLuc
shKdm5b
Elect. potential (Å)
−70
Center
+70
B
C
-1 nucleosome
downstream shift (+)
D
upstream shift (-)
E
+1 nucleosome
downstream shift (+)
Slide (Å)
F
G
-1 nucleosome
downstream shift (+)
H
upstream shift (-)

Figure S5

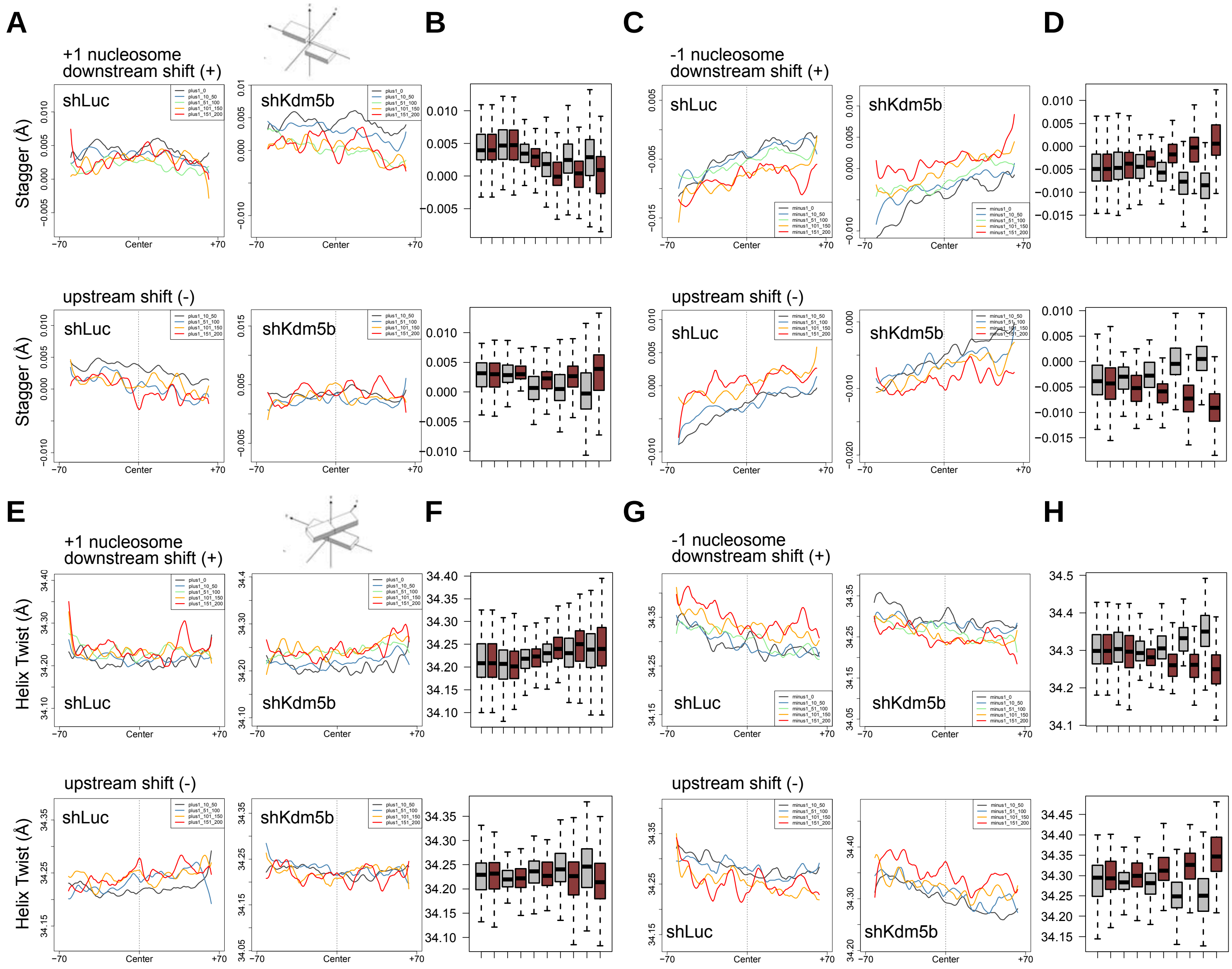
A
+1 nucleosome
downstream shift (+)
Stagger (Å)
shLuc
shKdm5b
upstream shift (-)
B
C
-1 nucleosome
downstream shift (+)
D
E
+1 nucleosome
downstream shift (+)
Helix Twist (Å)
F
G
-1 nucleosome
downstream shift (+)
H
Center
−70
+70

Figure S6
